# Supplementary material for: "I told myself, be bold and go and test": Motivators and barriers to HIV testing among gay, bisexual, and other cis-gender men who have sex with men in Ghana ‒ West Africa
Source: PLOS Glob Public Health. 2024 Jan 11;4(1):e0002231. doi: 10.1371/journal.pgph.0002231 (PMC10783711; doi:10.1371/journal.pgph.0002231)
Supplement: Attachment — Submitted filename: Response1GPH.doc [file pgph.0002231.s001.doc]

The Editor,

PLOS Global Public Health

**03/09/2023**

Dear editor

**Response to review correction; PGPH-D-23-00411**

We have initiated the following response to your correction request

1. We have attached the original IRB approval from Yale University, the PI and current corresponding author were affiliates of Yale University at the time of implementing the study. Other supporting IRB numbers for other co-investigators were also provided in the submission
2. As a study conducted as part of a clinical trial, we have completed the Clinical Studies Checklist recommended. We also attached the protocol for that details the process of the clinical trial implementation and reporting for you review. It has also been cited on page 5 line, 113.
3. We provided a sentence as expected in item 2, to show that we do have access to harmful information of participants on page 7, line 140 to 142.
4. We did not fill the additional form on observational studies as this was part of the clinical trial process, however, a review of the components seem consistent with contents of this manuscript when applicable.

We appreciate your consideration of this manuscript for your journal and look forward to hearing from you soon.

Sincerely
