# Supplementary material for: "I told myself, be bold and go and test": Motivators and barriers to HIV testing among gay, bisexual, and other cis-gender men who have sex with men in Ghana ‒ West Africa
Source: PLOS Glob Public Health. 2024 Jan 11;4(1):e0002231. doi: 10.1371/journal.pgph.0002231 (PMC10783711; doi:10.1371/journal.pgph.0002231)
Supplement: Attachment — Submitted filename: Response to Reviewer10.12.docx [file pgph.0002231.s002.docx]

Response to Reviewer

**"I told myself, be bold and go and test": Motivators and barriers to HIV testing among gays, bisexuals, and all other men who have sex with men in Ghana – West Africa**

Reviewer 1

If you want to cite a prior paper (authored by me!) that uses similar qualitative methods to reach quite similar findings among men who have sex with men in New York City, please see Kobrak, et al., 2022: [https://pubmed.ncbi.nlm.nih.gov/35536518/](https://urldefense.com/v3/__https:/pubmed.ncbi.nlm.nih.gov/35536518/__;!!MkQhnT5yHH0MPvOc00c!WfQaj3xpPe3jNflO_18zJXaz0JVvBKjndQepMmK1zbYZTclAaSJj8OD87O5hhnzYZ8-CrdEaSmGokxFfg3gMi9q8QGlNg1RNrw$)

**Response:** Thank you for recommending the article for readership; it shows how similar issues occur across the population in different settings. It was also helpful in making a case for the study's limitations, as the two papers observed similar limitations.

The abstract, the beginning of the Results, and the discussion repeat a summary of the same findings.
**Response:** We have reviewed the discussion and the beginning of the results section to make them more summarized instead of stating everything stated in the summary.

"There is limited research on the of GBMSM or into the factors that motivate or hamper HIV testing behaviors among GBMSM in Ghana."
- Are you only looking at men's HIV prevention experiences? How about those sexual or social experiences that relate to stigma and can affect testing?

**Response:** We used factors as a broader statement to cover stigma social and individual-level factors. We have rewritten this section to reflect the entire focus of the paper.

- "Gays" and "bisexuals" sound odd in U.S. English

**Response**: We appreciate the thoughts on terminology in the U.S. context. In our global context, our experience is that the use of terminologies is quite changing, and people have different thoughts about the appropriate terms to use. We prefer to use gay, bisexual, and other cis-gender men who have sex with men to address those who identify as gay or bisexual and others who do not in our context, as it is prevalent.

- In U.S., standard term is "assigned male sex at birth"

**Response**: Revised

"To assess the feasibility, acceptability, and potential effect size of a multi-level intervention to reduce intersectional HIV, gender nonconformity, and sexual stigma and to increase HIV testing among GBMSM in Ghana."
Or maybe instead, "Stigma related to… intersectional HIV."

**Response:** Revised

- "GBMSM disproportionately account for new HIV infections" seems inexact and maybe even a bit stigmatizing; maybe ""disproportionately acquire HIV" or "experience a disproportionate burden of HIV."

**Response:** Revised

- Clarify if IDIs are with individual men

**Response**: Yes, we confirm the IDIs conducted among ten men. We have edited that section a bit to make it clearer.

- Could make content and language more concise and avoid imprecise passive voice constructions: "While HIV testing practices are slowly increasing among GBMSM in Africa,[2, 19] there is a need to understand the factors the affect HIV testing behaviors for this vulnerable group, and to begin to address such factors" ◊"Further increasing HIV testing among GBMSM in Africa[2, 19] requires that we understand and address factors that can affect men's HIV testing practices."

**Response**: We have read through the manuscript and tried to be as precise as possible

- This method and framework needs explanation, and is unclear without reading the citations: "Recognizing the centrality of stigma in HIV testing practices, this study employs the Health Stigma and Discrimination Framework (HSDF) to unpack GBMSM's experiences across the socio-ecological model.[20] HSDF posits that the stigmatization process unfolds across the socio-ecological spectrum in the context of a health condition, such as HIV care, and ultimately impacting uptake of testing, adherence and treatment, incidence, morbidity and mortality among affected groups [20]."

**Response**: The framework is explained and embedded throughout the introduction.

Abstract

"infrequently often"?
**Response**: Revised

Maybe say "perceived stigma at Healthcare facilities"
**Response**: Revised

Define acronyms at first use

**Response**: Revised

introduction

Lots of data from another study: "For instance, condom use was higher when GBMSM had vaginal sex with women, (rel. f. = 0.75) than anal sex with men (rel. f. = 0.50) [14]. About 40% of did not use condoms during first sexual encounters, and even less (38%) continuously used condoms with sexual partners [14]. These increased risk behaviors and high HIV prevalence among GBMSM shows the need for increased HIV testing [10-16]."
**Response**: Revised

Aims: Focus on stigma?
**Response:** Stigma is part of the barriers, although it seems to be the main barrier; since we have other issues, we decided to capture everything broadly.

Method / Design / Data Analysis

You describe coding in detail. To complete the methodological description, could you add how you selected the coded content to include in the article?
**Response**: Added some details

**Results**
Some of the barriers and facilitators very succinctly described in quotes could perhaps benefit from longer quotes or additional narrative that more fully describe the participant's thinking and situation.
- For example, here is a great, counter-narrative quote that is quite long and gives the reader a sense of the participant's thinking: "Let me say, I am not interested in testing or I have never tested or nothing because as I said I work in the health sector and my philosophy in life is that, all die be die cos working in the health sector and looking at the things that are killing people in Ghana, HIV is not top 10 killer disease… I have never tested and I don't think I will ever test."
**Response**: We have added some contextual explanations to increase understanding of the quotes in the context of questions asked

I wondered if the quotes were directly from transcripts of interviews and focus groups. For example, do men in Ghana routinely refer to themselves as "MSM" as in quotes? e.g., "Once you have accepted you are an men who have sex with men…"; "As a guy or as an GBMSM having multiple sexual partners…"
**Response**: Yes, the quotes are directly from the focus groups, they don't say GBMSM, though. They either say gay, bisexual, or MSM. The GBMSM used in quotes is a typo when we used the replace option to ensure all terms are consistent; that is fixed now.

The introduction refers to "As such, there is lower use of prevention tools such as condoms, lubricants and HIV testing, and in some cases, limited adherence to medication."
- In the results, I saw limited results about the specific ways men have sex with other men that may put them at risk of HIV acquisition or influence their approach to HIV testing – e.g., few mentions of whether men use condoms or PrEP.
- Are there other findings on this that it would make sense to add?
- Is PrEP widely available to men in Ghana?

**Response**: For this paper, our scope concerns testing only. If the content is not on either a testing experience, barrier, or promoter of testing, they do not meet the scope of the paper. However, other papers published or under review from the dataset cover PrEP, medication adherence, and other factors affecting GBMSM in the country. Also, PrEP has been introduced in Ghana and is not widely available, but LGBTQ-specific providers have outreach to provide for the population. We cover these in our other papers.

Maybe explain a bit the importance of "treating them equally" i.e., did participants find that a lack of negativity or discrimination towards gay or femme-presenting men give them positive feelings that made testing easier?
**Response**: Yes, correct and revised

This seems like a very important quote about how to understand or encourage HIV testing, but I think it needs explanation: "I think the very best thing that could happen to someone who is MSM is to test for HIV like in very simple and short words. "
**Response**: We have reviewed all quotes and provided more context where we identified they were not clear enough

"HIV as a disease was usually historically associated with immoral behaviors, as a punishment for deviant behaviors related to sex or contracted by only certain groups such as MSM or commercial sex workers."
The effect of peer educators and testing providers promoting positive messaging about HIV testing seems so important. How did this work come about? Who built these programs?
**Response**: Peer educator services are implemented by Researchers, and HIV outreaches by private entities specifically targeting men who have sex with men. As seen in some quotes, these services sometimes come with incentives.

Organization: Perhaps, given the overlap between the Results sections
- Heading 2: Could call it "Other Motivators and Facilitators of HIV testing"
- Heading 3: Understanding the risk associated with sexual behaviors such as transactional sex served as a motivator for testing. But what followed did not describe experiences of transactional sex
**Response**: We reviewed the entire results section, moved quotes that were not applicable to areas where they applied or created subheadings to illuminate certain points, such as sex work. Now we have three main headings: one about experience at the time of testing, one for motivators and facilitators, and the last one for barriers. Under each, we then have the subsections.

Maybe indent quotes to avoid having to mark quotes inside quotes
**Response:** Correction effected.

Discussion

For the discussion could expand on the National HIV context: I was wondering, do men understand HIV as a disease that can be effectively treated? Has the situation changed in Ghana in recent years? Is HIV treatment now more widely available? Do men see their peers on HIV treatment thriving? (I see in discussion: "thus limiting the scope to testing and no other aspects of care")
**Response**: In the context of SSA, it's already established that treatment is widely available, but the main issue that affects HIV prevention and care is stigma. Hence, as seen in this study, we discussed that friendly healthcare providers increased testing. We did not consider discussing treatment and other aspects of care because they are beyond the focus of this study, and we cannot necessarily support those discussions with our current findings. Nonetheless, the implications of these findings affect all aspects of care, and most of the studies cited cut across these care areas. Therefore, based on this comment, we highlighted a few areas where these are applicable. We still limited our discussion to maintain the focus of the paper. We collected information on other aspects of care and have submitted them as a part of our series of publications from the current data set. Also, based on the findings, we discussed how understanding of HIV informed testing interests or experiences.

LGBTQ acceptance context: Any sense of whether there is growing acceptance among the public and the professional medical class in Ghana towards gay and femme men?
**Response**: The results show that this is mixed; while some providers were friendly, others were not so as friendly. We cited our current findings on interventions to address stigma at the healthcare facility level. At the community and national levels, gays and feminine men are highly stigmatized, and there is an ongoing law under consideration in Ghana's parliament to further criminalize same-sex intercourse. We have expanded the discussion to highlight this issue. We also mentioned it in the introduction.

This statement doesn't seem to match the experience of many of the men motivated to test: "As explained by the HSDF and previous findings, participants in this study internalized the stigma and begin to develop fear for HIV, thus experiencing stress during HIV testing, and or avoid testing in its totality [10, 23, 28]."

**Response**: In participant accounts, some indicated not using certain facilities due to stigma, they also experienced stress, etc. We probably overstated this, so we adjusted the sentence to show precisely what the participants mentioned.

**Reviewer 2**

Abstract:
Several abbreviations are used but not spelled at. Please write the following in full at first use: SSA, GBMSM, IDI, FGD.

**Response:** Revised

The sampling approach is described, but there is no mention of the study design.
**Response:** Revised

Stating that something happens infrequently often is contradictory and confusing.
**Response**: Revised

Main text: Revised
Please report all abbreviations at first use.

**Response**: Revised

It is unclear what "rel.f." refers to. See line 64.

**Response**: removed from the text, but it meant relative frequency.

How was the sample size determined? Did you stop at data saturation?

**Response:** This was part of a larger study described, so we based the sample size for the focus group discussion, which was 8 to 12 per group. Saturation was reached in the discussion, but we observed some participants who live with HIV were not as vocal. Hence, we also used the ten interviews among those living with HIV mainly for additional clarity. We limited the descriptions here because we published the entire protocol and implementation process and cited it in this paper.

Why were both indepth interviews and focus groups needed?

**Response**: We indicated IDIs were with those living with HIV. This was to allow them a private space to open up about their experiences. Focus groups did not consider HIV status and did not discuss those aspects. Much of the discussion on care and life with HIV is reported in another paper.

It would be helpful the highlight the themes identified by underlining them or using italics as allowed by the journal.

**Response:** Revised.

Consider putting each quote on a new line. This improved readability.

**Response**: Revised.
